# Current Trends in Computational Quantum Chemistry Studies on Antioxidant Radical Scavenging Activity

**DOI:** 10.1021/acs.jcim.2c00104

**Published:** 2022-04-18

**Authors:** Maciej Spiegel

**Affiliations:** Department of Pharmacognosy and Herbal Medicines, Wroclaw Medical University, Borowska 211A, 50-556 Wroclaw, Poland

**Keywords:** Antioxidants, density functional theory, computational chemistry, electronic structure, weak interactions, kinetics, thermochemistry, QM-ORSA

## Abstract

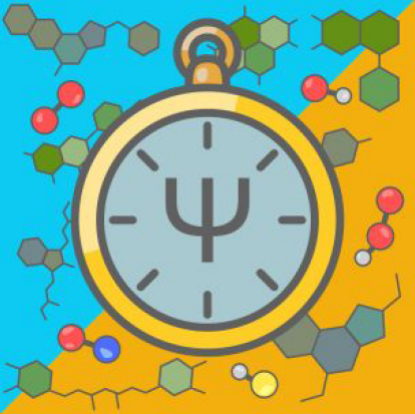

The antioxidative nature of chemicals is now routinely studied using computational quantum chemistry. Scientists are constantly proposing new approaches to investigate those methods, and the subject is evolving at a rapid pace. The goal of this review is to collect, consolidate, and present current trends in a clear, methodical, and reference-rich manner. This paper is divided into several sections, each of which corresponds to a different stage of elaborations: preliminary concerns, electronic structure analysis, and general reactivity (thermochemistry and kinetics). The sections are further subdivided based on methodologies used. Concluding remarks and future perspectives are presented based on the remaining elements.

## Introduction

Free radicals play a crucial role in the maintenance of homeostasis by participating in a range of physiologically relevant processes such as immune response and intracellular communication. Nonetheless, whatever the case may be, their uncontrolled accumulation is not favorable. The energy they possess as a due to the unpaired electron on the valence shell or excited state cannot be efficiently neutralized by intracellular antioxidant defense system and is instead transferred to the biologically important targets like lipids, carbohydrates, proteins, and DNA strains. In consequence, these structures degenerate, leading to the development of severe malfunctions resulting in illness such as diabetes, atherosclerosis, Alzheimer’s and Parkinson’s diseases, or tumor growth.^1,2^

The expanding awareness of the dual nature of radicals has prompted a surge in interest in compounds that can decrease elevated levels of reactive oxygen, nitrogen, and sulfur species, thereby preventing their harmful activity. These substances, known as *antioxidants*, are a heterogeneous group of molecules able to reduce oxidative stress in different ways. They are classified as follows^3^

- Type I: chain breakers, which interact directly with radicals by creating species that are more stable and less hazardous to cells than the former ones, thus terminating chain reactions and preventing oxidation of biological targets.
- Type II: preventers, for which, however, a unified mechanism of action is not specified, but it does not include interactions with radicals. Among known activities of that type are metal chelation, particularly iron and copper, which participate in the Fenton reaction, as well as regulation of enzymes responsible for radical formation or those directly involved in oxidative stress development.^1,2,4^ Compounds capable of regenerating biological antioxidants^1^ or absorbing UV radiation^5^ are also included in this category.
- Type III: substances that effectively repair oxidatively damaged biomolecules.^1^

However, because most antioxidants exhibit multiple types of activity at the same time, such categorization is often artificial. It is better to do so on the basis of their chemical structure or origin.

**Figure 1:**
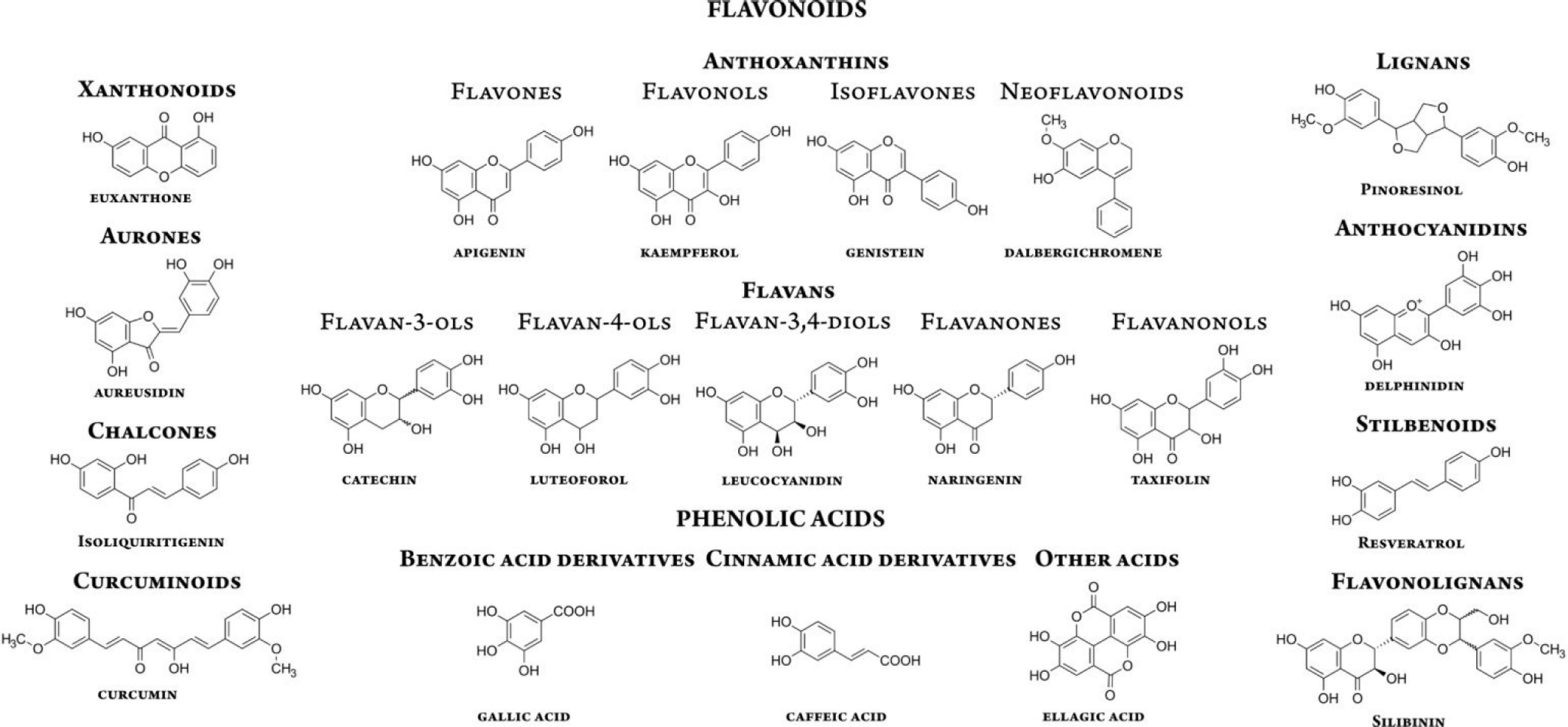
Some of the most commonly distinguished subtypes of dietary antioxidants and their representative examples.

Phytochemicals are plant-derived compounds that are plentiful in many herbs and commonly consumed plants such as beetroot, high in betalains;^6^ tea, rich in catechins;^7^ or grapefruit, abundant in flavanons.^8^ This family can be further subdivided into flavonoids,^9,10,19−28,11,29−37,12−18^ phenolic acids,^38−47^ lignans,^48^ aurones,^49^ chalcones,^49−51^ curcuminoids,^52,53^ anthocyanidins,^54−56^ stilbenoids;^28,57−59^ anthraquinones,^60−63^ glucosinolates,^64,65^ alkaloids,^66,67^ coumarins,^68^ terpenes and terpenoids,^68−72^ and others^41,73,82−86,74−81^ (Figure 1), all of them being extensively studied with computational quantum chemistry methods, as evidenced by the number of recent scientific findings. Albeit, plants are not the only source of antioxidants —substances with promising antiradical activities have also been found among common drugs,^87−92^ biological substances,^93−102^ and their metabolites.^59,93,98,100,103−105^ All of the listed substances are being heavily modified in an effort to identify derivatives with an improved safety profile and enhanced radical scavenging potential.^23,36,102,106−114,46,115−124,83,125,86,87,89,94,96,101^

Finally, completely novel structures are proposed and investigated on the basis of recognized pharmacophores: phenolic units,^126−132^ five-heterocyclic rings,^133−139^ quinoline backbones,^140−142^ and other moieties.^143−146^ Importantly, despite their diversity, chain breakers share similar reactivity patterns and mechanisms, allowing common theoretical approaches to be used to study any of them.

This review outlines currents trends in “Type I” activity research in a straightforward and methodical manner. The first section deals with preliminary concerns, such as selecting the appropriate level of theory, the solvation model, and the initial structures. Following that, the topic of electronic structure examination is discussed in light of the methodologies documented in the literature. The majority of this work is devoted to thermochemistry and kinetics research, which are the most important for comparing computationally produced results with experimental data. Finally, remaining issues are highlighted and perspectives on the subject provided.

Before proceeding, it is important to note that while this review specifically mentions hydroxyl groups any other residue in which a hydrogen atom is bonded to a highly electronegative atom, such as nitrogen in an amino group or sulfur in a thiol group, can also be considered as the one that may participate in antioxidative activity.^46^

## Preliminary Concerns

The level of theory chosen, which describes the electronic structure of the molecule *as itself*, and the solvation model, which adjusts the system’s electron density cloud to minor perturbations caused by solvent molecules, are two key components influencing chemical behavior that must be taken into account from the beginning of the studies.

### Functional and Basis Set

It is frequently advantageous to obtain results that precisely resemble experimental data while needing the least amount of computational time. However, this seemingly easy task is burdened with two fundamental issues: (1) With so many functionals and basis sets available, choosing a level of theory satisfying this condition is difficult. (2) The lack of reference data against which theoretical findings may be compared casts doubt on the latter. Although high-end methods such as CCSD(T)/CBS guarantee quality of the outcomes, this solution is inapplicable for routine computations due to the significant uptake of resources.

**Chart 1:**
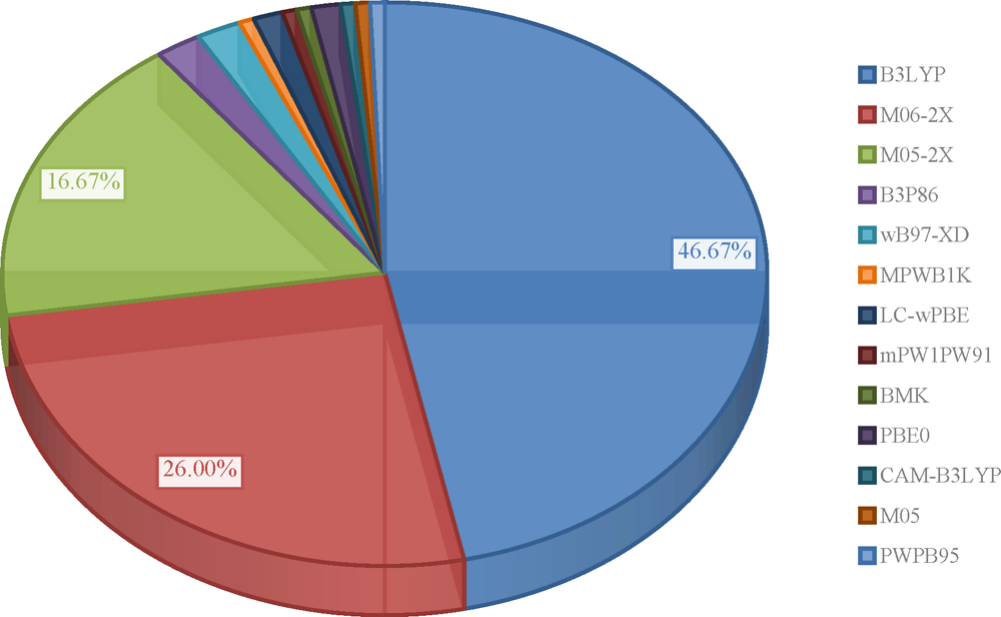
Share of Functionals in Articles Published in the Last Five Yearsa

As shown in Chart 1, there is currently a trend toward the use of density functional theory (DFT) methods, with B3LYP^147,148^ (∼47%), M06-2X^149^ (∼26%), and M05-2X^150^ (∼17%) being picked the most commonly. Restricting to any of them is advantageous because it provides researcher a plethora of datapoints the comparison with validate the results in a greater degree than it would happen if was done against outcomes obtained by completely differently constructed functional. Moreover, other researchers following the given reasoning would more likely cite the paper that use common level of theory since it enables to compare their own theoretical results with those provided by other researchers.

a Plotted on the basis of refs (9)–^146^.

Given their newness, it is visible that these two Minnesota functionals are gradually displacing B3LYP since their release. This could be a result of their superior performance in estimating thermochemistry, kinetic and noncovalent interactions of nonmetal elements, as well as energies of reactions involving free radicals, which they predict extremely close to actual data, as claimed by the developers^149−151^ and continue to be substantiated by independent scientists, either in the course of original research^39−41,45,77,84,87,129^ or in benchmarks.^152−154^ On the other hand, several researchers who used both B3LYP and one of Truhlar’s global hybrids noted that while B3LYP tends to underestimate energies there are no substantial differences and the reactivity patterns hold when the same basis set is used.^60,64,95,123,127^

Although the reasons for selecting certain functional are usually covered in the manuscript with details, the arguments for choosing “*this, but not that*” basis set are nearly always glossed over. This bad habit have its consequences in the performance of computations. Increasing the number of basis functions is known to increase task processing time, and it has recently been established that those from the Dunning’s and Ahlrich’s families are particulary vulnerable.^153^ Furthermore, whereas functionals should be kept constant throughout the research, the basis set does not, namely while one can be used for electronic structure investigations, other may be applied for thermochemistry, which allows maneuvering them in order to obtain the best results at the lowest possible cost.

**Chart 2:**
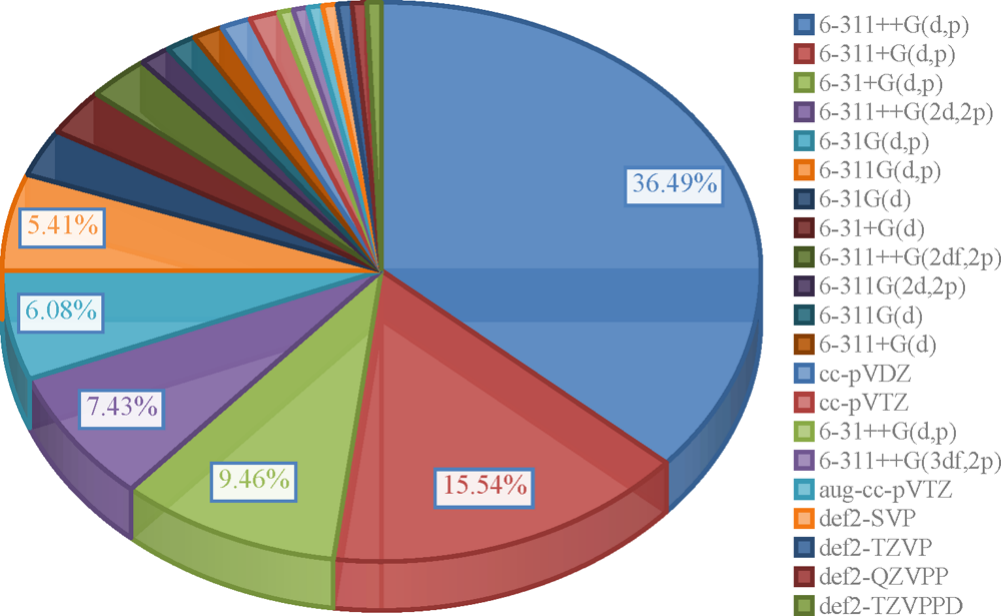
Share of Basis Sets in Articles Published in the Last Five Yearsa

As a result, Chart 2, which depicts the percentage of basis sets used for thermochemistry computations, is much more divided than the previous one, with Pople’s leading the way. A particularly good observation is that combining any of them with one of the three most commonly employed functionals presented in Chart 1, appears to have a little influence on the results as evidenced in the following examples. The findings of Shammera Ahamed et al.^79^ (B3LYP and M062X combined with either 6-31+G(d,p) or 6-311++G(d,p)) and Mendes et al.^12^ (B3LYP, LC-wPBE, M062X, and BMK in 6-311G(d,p) and 6-311+G(d,p) bases) show that despite the augmentation of basis set with another diffuse function or inclusion of next function describing valence shell, the values of reactivity indices found in the gas phase change only slightly, regardless of level of theory used. In their other study,^26^ the authors demonstrated that shifting from 6-311G(d,p) to 6-311+G(d,p) changed the ionization potential values by less than 4 kcal/mol independently of the environment (gas phase, water, methanol, ethanol, *n*-hexane). Similar results were observed in the investigations of Bakir et al.^146^ (B3LYP/6-31G(d,p) and B3LYP/6-311++G(2d,2p)) and Santos et al.^60^ (B3LYP/6-31+G(d,p), B3LYP/6-31++G(d,p) and B3LYP/6-311+G(d,p)), both of which were conducted in gas and water. On the other hand, the differences in ionization potential and electron transfer energy associated with ion formation in water milieu were found to be significantly smaller in the case of B3LYP/6-311++G(d,p) outcomes when compared to those obtained at B3LYP/6-31+G(d,p) level of theory.^79^

a Plotted on the basis of refs (9)–^146^.

Moreover, although the presence of polarization functions is undeniably important for determining the energy of highly polarized bonds, the role diffusion functions, which are widely regarded as necessary for accurately modeling electron clouds in ionic systems and radical involving pathways, must be addressed more thoroughly. Still, the recent research^153^ has shed new light on that issue for it was discovered that using 6-311G(d,p) for overall antioxidant studies produces the best results in terms of both the accuracy between theroetical outcomes and experimental values, as well as computational resource uptake. Further confirmation on that indulging issue and more detailed studies on the role of the basis set are welcome.

It is worth noting that open shell computations are hampered by the possibility of spin contamination.^155^ This is because the resulting wave function is an artificial mix of spin states rather than an eigenfunction of total spin, ⟨*S*^2^ ⟩. In an ideal system, ⟨*S*^2^ ⟩ equals 0.75 for singlet and 2.0 for triplet
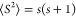
1where *s* denotes the number of unpaired electrons divided by half. Other values are acceptable as long as they deviate by no more than 10%.^10,109,152^ Greater ones indicate the presence of higher spin states, which may alter the energy or geometry, just like the population analysis outcomes, resulting in biased conclusions; such structures should not be considered for future research. Spin-restricted open shell computations may be a solution in those cases, but they consume more resources than unrestricted ones and may still produce incorrect energies of unpaired electrons due to the absence of dynamical correlation caused by the vanishing of spin polarization.

### Solvation Model

The physiological media in which antioxidants play fundamental biological roles are body fluids and the lipid bilayer of cells membrane. As a consequence, most experiments are conducted in water or in a nonpolar environment, that computational elaborations must account for. The effect of a polar solvent is a fundamental tenet of theoretical investigations because it distorts a molecule’s electron cloud due to electrostatic polarization interactions, affecting the shape of the potential energy surface and chemical activity.^3,20,28,130,156^ Furthermore, if the reaction pathway involves proton or electron detachment, the solvation is a known to be a driving force that eases the process and makes it more feasible than it would be in a nonpolar medium.^50,116^ That is why it is also important for properly modeling dissociation related processes as is stressed later in the text.

For the time being, three methods are employed to incorporate solvent effects: implicit (through a homogeneously polarizable medium), explicit (by solvent molecules), and a combination of these two. The integral equation formalism variation of the polarizable continuum model (IEFPCM, often referred to as just PCM),^157^ conductor-like polarizable continuum model (CPCM),^158,159^ and solvation model based on density (SMD)^160^ are examples of continous solvation models that are implemented in a majority of quantum chemistry software. They are also the most frequently used because they are burdened with a much lower computing cost than implicit or combined approach.

However, the fundamental disadvantage of implicit models is complete negation of intermolecular hydrogen bonds, which are often essential for proper simulation of antiradical activity^93,103,144^ especially when abundant as in the case of capsaicin.^84^ A mutual competition between intramolecular and intermolecular hydrogen bonds is also observed as a relevant factor modying hydrogen atom transfer proclivity.^47^ The modeling of ^•^OH and ^•^OOH radicals, which are of primary interest in antioxidants research, is an excellent example of how the explicit water molecules can influence the results as well. Accordingly to Pérez-González and Galano,^125^ adding the first water molecule considerably changed the rate constants of the ^•^OH/^–^OH path, but adding more or repeating the procedure for the ^•^OOH/^–^OOH path resulted in no significant change. The fact that their charge is concentrated on a single exposed heteroatom^161^ is important here.

The application of a homogeneously polarizable medium is a particularly viable method of modeling solvents having large structures. For example, representing the enormous membrane lipid chains would be a time-consuming and inefficient effort. Instead, one of the most common approaches found in the literature is to do that implicitly, by using the largest available aprotic solute, for example, pentyl ethanoate.^12,100,107,120,132^ To account for the cage effect, which manifests as the loss of entropy of any chemical reaction with a molecularity of two or greater, and improve results, the Okuno’s corrections^162^ and Benson’s free volume theory^163^ can be applied (eq 2), as demonstrated.^40,41,104^ The corrected Gibbs free energy, Δ*G*_solv_^FV^, is expressed then in a form of
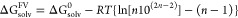
2where *n* represents the molecularity of the reaction, and Δ*G*_sol_^v0^ is a Gibbs free energy in solvent, *R* a gaseous constant, and *T* the temperature^164^

### Initial Structure

Let’s refere a thorough conformational studies^156,165^ that were performed on a collection of quercetin structures with variable planarity and intramolecular hydrogen bond counts. These two structural features are known to account for antioxidative activity of flavonoids, and so considerable differences have been found among them. This is not the only case, for similar geometry–activity relationships have been pinpointed also in other studies.^60,91,107,134^ This emphasizes the role of selecting the appropriate conformer for the study as a critical first step in theoretical elaborations.

Molecular dynamics (MD) simulations are most effective for producing excellent starting structures. Different approaches can be undertaken to run MD calculations, such as using software that has implemented molecular mechanic potentials and thus allows for the direct conformational search procedure^166−168^ or dedicated ones designed for more demanding studies, like GROMACS,^169^ Amber,^170^ or CHARMM,^171^ for which the molecule under consideration must first be parametrized. This can be done with either the proposed protocols^84^ or available webservices the most well recognized of which is probably CharmmGUI,^172^ although AutomatedTopologyBuilder (ATB)^173^ should also be mentioned.

CharmmGUI has an advantage over ATB in that it has a user-friendly interface and instantly generates a complete set of files that can be submitted for molecular dynamics, whereas ATB only produces force field, structure, and topology data, leaving the user to prepare the remaining files. CharmmGUI, on the other hand, predicts topology using CGenFF,^174^ which elucidates it through bond perception and atom typing, while ATB processes input using DFT or semiempirical techniques depending on the size of the system. After all, it is the user’s expertise that determines which path to take.

MD frequently generates a large number of molecules, whilst upcoming quantum chemical studies should only consider the most populated states. One of the first filters used to get rid of the undesired structures is the geometric clustering algorithm^175^ which, in simple terms, groups conformers based on their structure or kinetics. If there are multiples of them, optimization at the appropriate level of theory followed by Maxwell–Boltzmann distribution analysis may be a viable approach for removing superfluous structures, particularly those with molar fractions less than 0.1%. This threshold is proposed^46,101,112^ because a small energy difference between conformers could indicate an interconversion process: rotatability of OH groups^165^ or side chains,^84^ bonds deformation due to keto–enol tautomery,^21^ shift in E/Z-conformers equilibria,^133^ and bending of dihedral angles.^16,20,28,33,50,67,128,165^ They can all modulate hydrogen bonds, electrons cloud delocalization, and polarizability, as well as radical accessibility to specific sites of an antioxidant. Furthermore, the molar fraction cannot be too low if the compound is desired to pass biological barriers.^46^

Two equations (eqs 3 and 4) can be used to evaluate the Maxwell–Boltzmann population *f* of a specific conformer *i*, in the set of *n* conformers
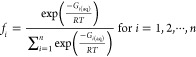
3and
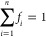
4where *R* is the gas constant, and *T* is the temperature. *G*_*i*__(aq)_, the aqueous phase Gibbs free energy of the *i*th conformer, can be calculated following eq 5
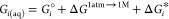
5where *G*_*i*_^*°*^ denotes a species’ gas phase free energy at a given temperature, Δ*G*^1atm→1M^ = 1.89 kcal/mol and reflects the shift in standard state from 1 atm (superscripted with °) to 1 M (superscripted with ∗), and Δ*G*_i_^*^ denotes a species’ aqueous solvation free energy.

### Deprotonation and Dissociation Constants

The primary activity of type I antioxidants is based on their reductant capacity, which is frequently, but not always^64,68,125^ linked to the hydrogen-donation capacity from one of their aromatic hydroxyl groups; thus, a simple conclusion can be drawn that the more of them, the greater shall be participation of hydrogen-related channels in overall radical scavenging, and so its viablity.^116^ However, because these residues are also weakly acidic, multiple species can coexist in a water environment at the same time. Their molar fraction is governed both internally by their chemical structure^82^ and externally by the pH of an environment.

If radicals are neutralized by mechanisms inaccessible to other forms, a seemingly small amount of one of them may be critical in accurately measuring scavenging activity. At the given pH, for example, it is possible that the antioxidants have already deprotonated all of the hydroxyl groups and thus exhibit only electron-related channels,^36,85,102,126^ whereas the studied radical is efficiently neutralized solely by formal hydrogen atom transfer. Similarly, with each subsequent dissociation, species are less likely to remove another proton, making routes that include its donation, e.g. sequential proton-loss electron transfer or sequential electron transfer-proton transfer, more energetically demanding; on the other hand, electron-donating processes are expected to occur more easily for them.

There are several methods that can be used to estimate dissociation constants and deprotonation pathways. The oldest ones, such as *direct*, *proton exchange*, *hybrid cluster continuum*, and *implicit–explicit*, rely on thermodynamic cycles and have previously been exhaustively discussed in the literature^176,177^ and are thus just briefly mentioned here.

**Scheme 1:**
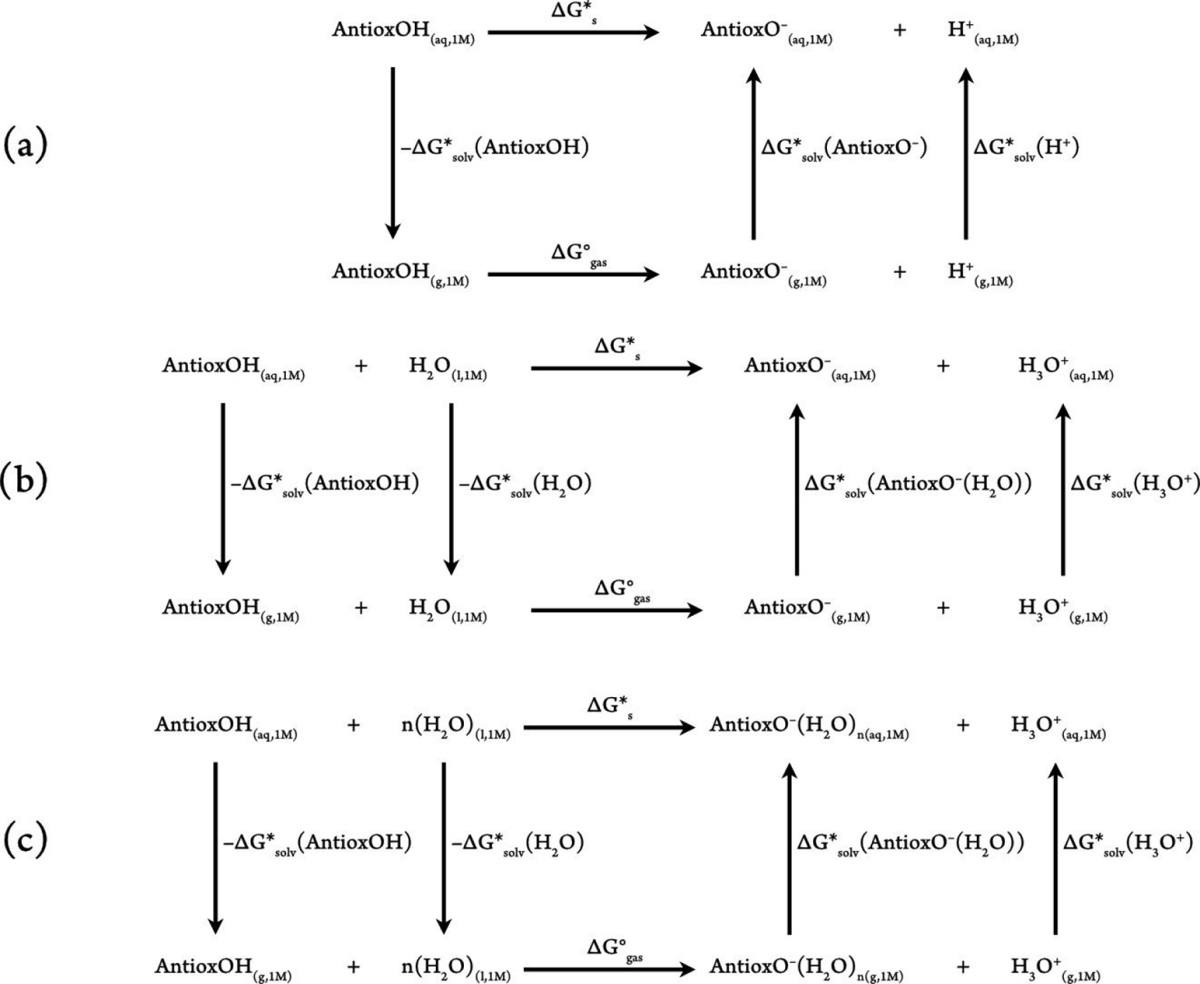
Various Thermodynamic Cycles Used to Calculate the Free Energy of Deprotonation

In the *direct* approach, an antioxidant may either (a) adhere to the Arrhenius theory and dissociate directly into anion and proton (Scheme 1a), (b) obey Brønsted–Lowry acid–base theory and react with a water molecule to form conjugated pairs of acid and base (Scheme 1b), or (c) react in the same way as the previous one but in a more sophisticated version in which the formed ion is also solvated by an arbitrary number of water molecules (Scheme 1c).

Regardless of the picked cycle, Δ*G*_s_^*^ represents the free energy of deprotonation, which is calculated using eq 6
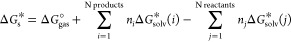
6

Then, p*K*_a_ is determined using a mathematical formula (eq 7)
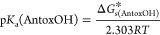
7

Despite the fact that one of the major drawbacks for this method is it requires proton or hydronium ion enthalpies, for which theoretical studies do not perfectly match, due to the impact of different solvation models used^178^ or methodology,^179^ it is still widely used^27,114,139^ owing to its simplicity. The question and recommended values of solvation enthalpies have been extensively elaborated by Marković and coworkers.^178,180,181^

Another way for determining pH is the *relative method* or *isodesmic method*. It is based on the proton exchange equilibrium between the acid of interest and the conjugated base of the reference acid and is represented by the following general chemical reaction
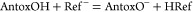


The acid–base pair of a reference chemical is defined as HRef/Ref^*–*^, and p*K*_a_ is computed in the same way as in the direct method but with a modified form of the previously supplied equation, given here as eq 8
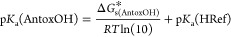
8

Although this method has been shown to produce accurate results,^58,69^ it requires HRef to be as structurally close to the HA as possible, and also the knowledge of the experimental value for p*K*_a_(HRef).

Among the newer approaches, the *parameters fitting method*(182,183) stands out not only due to its much simpler computation protocol but also the accuracy reported in the number of studies that have used it.^36,41,46,73,85,101,112,126^ It employs a linear regression model to determine p*K*_a_ values for hydroxyl, carboxylic, amino, and thiol groups in a water solvent. Mathematically, it is expressed by eq 9
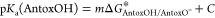
9where Δ*G*_AntoxOH/Antox^–^_^*^ denotes the difference in Gibbs free energy between the antioxidant’s conjugated base and the corresponding acid, and *m* and *C* are empirical parameters available for 20 different functionals, each in one of four Pople’s basis sets.

The former, however, is restricted solely to the water solvent. The solution is the *empirical conversion method*(184) presented recently. Although it does not allow for the calculation of p*K*_a_ values from scratch, the authors claim that it is useful for converting empirically determined dissociation constants in one solvent to any other with a little error.

Because both, the *parameters fitting method* and the *empirical conversion method*, were developed on strong experimental foundations, combining them in critical situations may not be such a bad idea. However, further testing is required to confirm this.

## Electronic Structure Investigations

### Intrinsic Reactivity Indices

A comprehensive study that covers the entire spectrum of antioxidative activity is difficult and time-consuming process. The intrinsic reactivity indices (Table 1) can be evaluated to obtain preliminary data that will guide further steps of the research. Although they do not consider the radical scavenged, the evaluation of preferred reaction paths of isolated species, identification of the most promising ones for a specific goal, and comparisons across antioxidants with similar chemical nature and modes of action is given by them.

**Table 1:** Names, Associated Reactions, and Explanations of Intrinsic Reactivity Indices

Name (typical acronym)	Related reaction	Brief description
Ionization potential (IP)	Antox(OH)_*n*_ → Antox(OH)_*n*_^+^ + e^–^	The ability to contribute an electron, which is interpreted as a willingness to oxidize itself. The lower the IP, the greater the likehood of antioxidant protection through electron transfer via electron donation. This is sometimes referred to as ionization energy (IE) in the literature.
Electron affinity (EA)	Antox(OH)_*n*_ + e^–^ → Antox(OH)_*n*_^–^	The ability to accept an electron, which can be interpreted as a desire to reduce itself. The lower the EA, the more likely antioxidant protection through electron transfer via electron acceptance.
Bond dissociation enthalpy (BDE)	Antox(OH)_*n*_ → Antox(OH)_*n*-1_O^•^ + H^•^	The amount of energy required to break the O–H bond during homolytic fission, which can be interpreted in the context of the radical’s stability. The lower the BDE values, the more active the corresponding −OH residue is in the hydrogen atom transfer mechanism and the more stable the radical formed.
Proton affinity (PA)/Proton dissociation enthalpy (PDE)	Antox(OH)_*n*_ → Antox(OH)_*n*-1_O^–^ + H^+^	The amount of energy required to break the bond during heterolytic fission, which can be interpreted as the anion’s stability. The lower the PA/PDE value, the more the corresponding −OH residue will be deprotonated. PA is defined as the inverse of the enthalpy change in a gas phase reaction between an electrically neutral chemical species and a proton to form the conjugated acid of the latter, whereas PDE is the deprotonation of a radical cation in any medium.

Notably, reactivity indices can be calculated vertically or adiabatically, that means with or without orbital relaxation. IP and EA are particularly important in Marcus theory for calculating the activation energy of electron transfer mechanisms and are thus mostly established in this context. The following equations (eq 10 and eq 11) are used to determine their vertical values
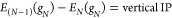
10
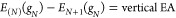
11

The adiabatic ones, on the other hand, are calculated as (eq 12 and eq 13)
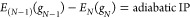
12
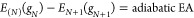
13where *E*_*N*_, *E*_(*N*–1)_, and *E*_*N*__+1_ denote the total energies of the *N*, *N* – 1, and *N* + 1 electron systems, respectively, computed at ground state geometries of (*g*_*N*_), (*g*_*N*__–1_) and (*g*_*N*__+1_) systems.

The Hammet sigma constant is one of the tools that draws from reactivity indices. It reflects the electron withdrawing or donating capacities of substituents connected to the aromatic moiety and thus can be applied to assess their impact on the intrinsic reactivity indices in a semiquantitative manner.^22,23,32,127^ They can be also used as a features of quantitative structure–activity relationship (QSAR) models, which numerically relate them and other descriptors to experimental data.^11,24,43^ Recently, an extensive paper on QSAR development and validation was published.^185^

### Frontier Molecular Orbitals

The energy and distribution of frontier molecular orbitals, specifically the highest-occupied molecular orbital (HOMO), a nucleophilic part of the molecule, and the lowest-unoccupied molecular orbital (LUMO), an electrophilic part of the molecule, can be directly linked to antioxidative activity.^186^ This is because Janak’s theorem^187,188^ states that the energies (ε) of HOMO and LUMO are related to ionization potential (eq 14) and electron affinity (eq 15), respectively, via the following relationship:
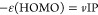
14
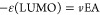
15

A molecule with a low HOMO eigenvalue is likely to be a poor electron donor, whereas a molecule with a low LUMO eigenvalue is likely to be a good electron acceptor. Analyzing their values allows for the initial elucidation of the antiradical activity. Although the majority of naturally ocuring radicals are electrophillic, and so the interaction between their SOMO and antioxidants' HOMO is of the greatest importance, there also exist nucleophilic ones, typically carbon-centered, which scavenging potential is subjected to the overlap between SOMO of the reactive specie and LUMO of the scavenger. In addition, visualizing HOMO allows for a prediction of which molecular site is more vulnerable to radical attack^28,50,116^, but presented later Fukui functions are more reliable option.

These approximations of orbitals egeinvalues as ionization potential or electron affinity, however, ignore electron correlation and are highly dependent on the method and basis set employed^27,52,123,153^. This poses further problems for they serve as the foundation for a slew of global descriptive parameters related to the electrophilic character of the species (Table 2)^189^ meaning their improper values may lead to further errors in the study and invalid conclusions. Given the importance of being as precise as possible, direct computations, are strongly advised. On the other hand, M062X/6-311G(d,p) level of theory has been found to approximate energies of frontier molecular orbitals with an error no greater than 0.5eV from the ones calculated in the direct vertical manner.^153^

**Table 2:** Names, Mathematical Formulations, and Descriptions of Indices Related to Frontier Molecular Orbitals Theory

Name (typical acronym)	Related formula	Brief description
HOMO–LUMO gap (HLG)	HLG = IP – EA	Represents the ease with which the electron in a molecule can be excited from HOMO to LUMO. The lower it is, the easier the electron migrates from one another, and the radical reaction proceeds more quickly because it is more kinetically stable.
Electronegativity (χ)	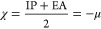	The general proclivity to attract electrons.
Chemical potential (μ)	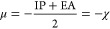	Indicates the direction of charge flow as well as the capacity to contribute or accept it. Electrons will migrate from high to low μ locations in a quantity proportional to changes in μ, with a corresponding stabilizing energy μ.^2^
Global hardness (η)	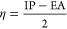	Measures the resistance to electron cloud polarization caused by a minor chemical disturbance or a change in electron number.
Global softness (*S*)	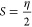	The ability to accept electrons. It is inversely proportional to chemical hardness.
Electrophilicity (ω)	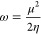	The ability of a system to acquire a partial charge. When two molecules are involved in a chemical reaction, the one with the higher value is considered the acceptor, while the one with the lower value is considered the donator. It is advised to be used to demonstrate the efficacy of electron donation in compounds with extremely low IP values.

To ensure the quality of vertical energies obtained, an electron propagator theory (EPT)^190,191^ and a partial third-order quasiparticle theory (P3)^191^ can be exploited. These theories lay the groundwork for the systematic inclusion of electron correlation in a one-electron model of molecular electronic structure. They have been shown to produce lower mean errors than any other open shell techniques when compared to experimental trial results^192^ and are now wiedly used.^46,101,112^ For the EPT estimations to be valid, the pole strength (PS) values must be greater than 0.80–0.85.^193,194^

### Electron and Hydrogen Donation Properties

To do quick and simultaneous comparisons of the relative electron-donating and electron-accepting properties of a set of species the donator-acceptor map (DAM) is a right choice.^195^ It is based on the assumption that in a simple charge–transfer model the response of a molecule submerged in an idealized environment that can either remove or donate charge can be represented by a quadratic interpolation for the energy as a function of the number of electrons.^196,197^ Therefore, DAM exhibits the antioxidant’s tendency in charge-related processes in terms of electron-donating (*ω*^*–*^) and electron-accepting (*ω*^*+*^) powers (Table 3).

**Table 3:** Names, Mathematical Formulations, and Descriptions of DAM-Related Indices

Name (typical acronym)	Related formula	Brief description
Electrodonating power (ω−)	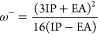	The ability of a chemical system to provide a fractional amount of charge. The lower the ω–, the more likely it is that the molecule will behave as an electron donor in weak interactions with other species.
Electron-accepting power (ω+)	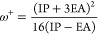	The ability of a chemical system to receive a fractional amount of charge. The greater the ω+, the more likely it is that the molecule will behave as an electron acceptor in weak interactions with other species.
Donor index (*R*_d_)	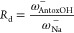	
Acceptor index (*R*_a_)	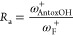	
Relative value of electron acceptance (REA)	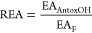	
Relative value of electron donation (RIE)	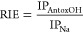	

**Figure 2:**
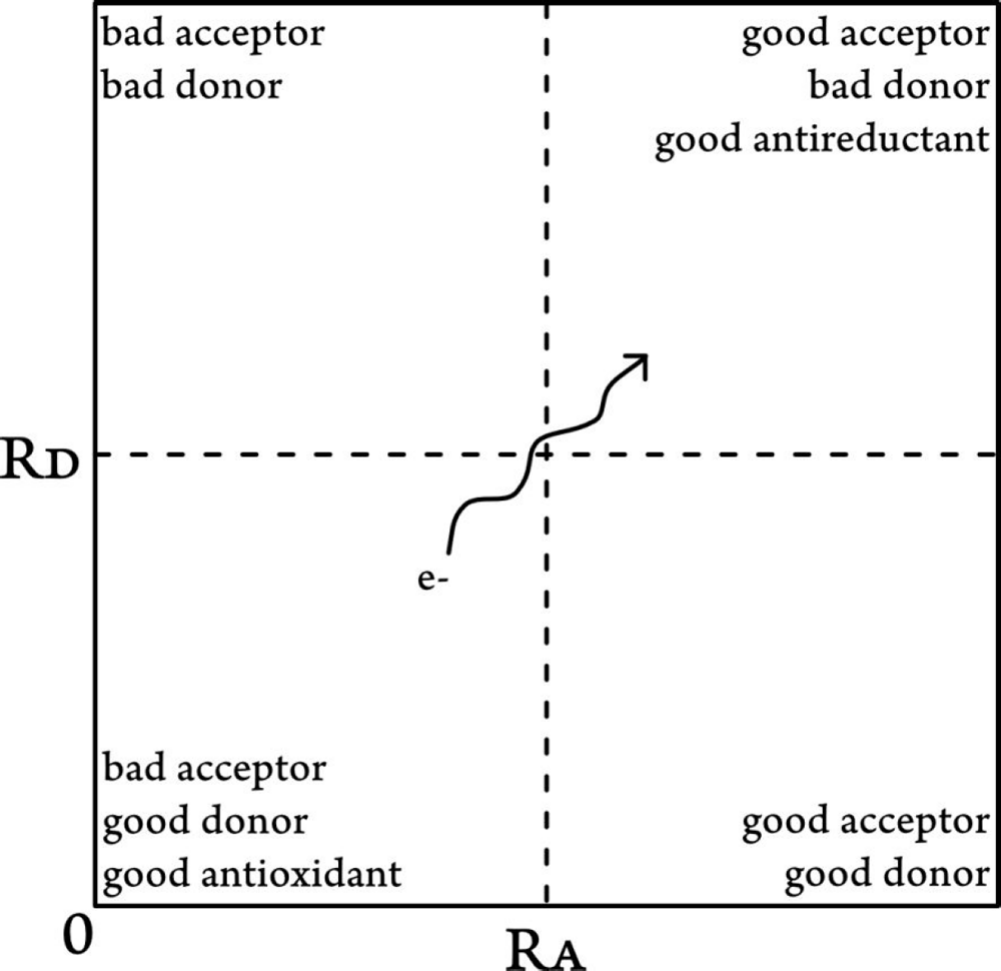
Schematic representation of donator–acceptor map.

The substance is classified into one of four distinct zones (Figure 2) based on precalculated donor (*R*_d_) and acceptor (*R*_a_) indices: (1) the excellent antiradical zone (lower right), where it is both a superior electron donor (small *R*_d_) and acceptor (high *R*_a_), (2) the worst antiradical zone (upper left), where it is both a poor electron donor (high *R*_d_) and a poor electron acceptor (small *R*_a_), (3) the good antireductant zone (upper right), where it is a fine electron acceptor (high *R*_d_ and *R*_a_) and thus an effective antiradical, and (4) the strong antioxidant area (lower left), where its good electron donor properties manifest (small *R*_d_ and *R*_a_).

*R*_d_ and *R*_a_ are valued for their electron-donating and electron-accepting powers, respectively, and are defined in relation to the electron-accepting power of fluorine (ω_F_^+^ = 3.40) and electron-donating power of sodium (ω_Na_^–^ = 3.46). F^–^ and Na^+^ are used as references due to their high electron-accepting and electron-donating capacities. ω_F_^+^ and ω_Na_^–^ can be calculated using experimental data.^32^ If *R*_a_ < 1, the substance is a poorer electron acceptor than F, and if *R*_d_ > 1, it is a poorer electron donor than Na.

Three of the four zones represent the intended antioxidative activity. In the above graph, the electron flow goes from species in the bottom left to species in the top right. When comparing substances, DAM helps to predict which will behave as stronger or weaker oxidizers or reducers,^85^ while also accounting for the effects of the environement.^62,71^ It is good to remember, there is a general trend of increasing electron-donating properties with each subsequent dissociation, so polyanionic species shall generally have lower *R*_d_ values than neutral of cationic forms.^50,91^

Full electron–donor–acceptor map (FEDAM), an improved version of DAM, has been developed to account for the nature of the interacting free radical, which can have such a significant impact that it can even invert the relative importance of the free radical scavenging mechanisms.^198^ FEDAM is based on the relative values of electron acceptance (REA) and electron donation (RIE) indices derived from vertical IP and vertical EA, again with Na and F atoms used as references. These are plotted on a map in the same way that DAM is but for both antioxidants and radicals. It has been used to evaluate antioxidant activity of melatonin, as well as its metabolites and derivatives.^101,105^

The electron- and hydrogen-donating ability map for antioxidants (eH-DAMA)^101^ is another approach to enhance DAM. It was designed to identify compounds that are good donors in both electron transfer (low ω) and hydrogen atom transfer (low BDE). It is similar to DAM, but the axes changes, so the Y axis corresponds to ω, while the X axis refers to BDE. As a result, the species in the left-bottom region are more likely to work in both directions, making them particularly valuable as radical scavengers, such as new sesamol^112^ or melatonin derivatives^101^, or modified p-coumaric acid analogs with neuroprotective activity.^46^ In most cases, parent molecules, oxidants, or reference antioxidants, e.g. Trolox, are included for comparison purposes.^46,112^

### Radical Attack Site

Frontier molecular orbital theory introduces a numerical method for investigating the reactivity of individual sites of molecule in three types of reactions. Fukui functions^199^ were proposed to represent the difference in electron density at a given point, ρ(*r*), as a function of the number of electrons, *N*, at a given external potential, υ(*r*) (eq 16). This is based on the notion that the optimal path for a reagent to approach the other species is the one with the highest initial fluctuation of the electronic chemical potential, μ.
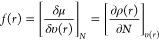
16Integrating over that equation for individual atoms in a molecule yields condensed Fukui functions (eqs 17–19), which are a more convenient way of predicting the reaction site than visualization of HOMO or LUMO orbitals. In general, the higher the value, the more reactive this position is to the specific type of attack. For an arbitrary atom A, these functions are defined as
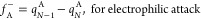
17
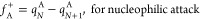
18
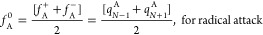
19where *q*_*N*–1_^A^, *q*_*N*+1_^A^ represent charges of (*N −*1) and (*N +* 1) systems obtained vertically from the optimized ground state geometry (*N*) with charge *q*_*N*_^A^. This ensures that the values are solely determined by the atom’s electronegativity and electron density location. At the same time, because of that the atomic charge values used to estimate condensed Fukui functions are heavily reliant on population analysis method.^124,200,201^ Given their varied formulations,^202^ the condensed Fukui functions may exhibit significant mutual discrepancies—even negative values^28^—but this has already been explained as a result of the small interatomic distances between atoms.^203,204^ So Atoms-in-Molecule tend to overestimate them;^204^ Natural Population Analysis is heavily influenced by the functional, and basis set chosen;^205^ and ESP derived charges, such as ChelpG, have nonsmooth geometry dependence.^200^ Also a solvent may also affect the final result.^124^ Some studies propose stockholder charge partitioning approaches in this purpose, particularly Hirschfeld analysis,^15,64,123^ since it yields values for which condensed Fukui functions correlate well with the expected data. In fact, a “dual descriptor” better illustrates susceptibility for electrophilic–nucleophilic attack,^206^ but these types of reactions are beyond the scope of antiradical activity studies.

### Redox Potentials

As it was already mentioned, primary activity of antioxidants is related to the direct reduction of radicals. Therefore, the viability of this process can be assessed in terms of electrochemical potentials.^207^ The Born–Haber thermodynamic cycle is one universal method due to the common pattern of reactivity, and a half-reaction describing that process is always denoted by
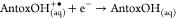
for which the standard reduction potential can be calculated using the Nernst equation (eq 20)
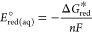
20where Δ*G*_red_^*^ denotes the standard Gibbs free energy of the reduction, and *n* is the number of electrons transferred and *F* the Faraday’s constant (23.06 kcal/molV). According to the Born–Haber cycle, Δ*G*_red_^*^ equals
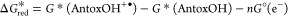
21with *G*°(e^–^) being free energy of one electron in the gas phase (−0.876 kcal/mol at 298 K). When comparing results to those obtained experimentally, the computed values must be reduced by an absolute potential of the reference electrode, for example, a standard iron electrode or a standard hydrogen electrode.

Notably, because certain compounds may be partially deprotonated at a given pH, and anions have a lower capacity to donate electrons than neutral forms, they also influence the overall reduction potential. As a result, the average can be calculated accordingly to eq 22
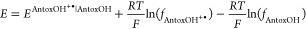
22where *E*^AntoxOH+•|AntoxOH^ represents the initial reduction potential, and *f*_AntoxOH^+•^_ denotes the population of the oxidized species that are reduced to produce the species with population *f*_AntoxOH_.

### Topology Analysis

Hydrogen bonds and weak interactions are important in stabilizing radicals or transition states rendering reactions to be more feasible, for example, by lowering BDE values.^50^ Bader’s Quantum Theory of Atoms in Molecules (QTAIM) can be used to quantify their strength^208,209^ and thus has been widely applied in antioxidants research.^31,58,62,103,122,130^ Despite the analysis’s complexicity, specialized tools such as the Multiwfn software^210^ eases it.

The nature of chemical bonds at bond critical points (BCPs) can be mathematically described by electron densities, ρ, and their associated Laplacian, ∇^2^ρ. The theoretical background is explained in detail in Bader’s paper;^208^ here, it will only be mentioned that BCPs of primary importance in such studies correspond to (3,–1) critical points, being the saddle points with a maximum of electron density in two directions of space and a minimum in the third, and that the number of BCPs must obey the Poincare–Hopf rule.^211^

The presence of a bond path between two atoms with a BCP in the middle is the first sign of a bond presence.^208,215^ A second criterion for defining it is that the values of ρ(BCP) and ∇^2^ρ(BCP) are positive, in ranges of 0.002–0.035 and 0.024–0.139, respectively.^215−217^ ∇^2^ρ(BCP) can be expanded as the sum of the eigenvalues λ_1_, λ_2_, λ_3_, obtained by diagonalizing the Hessian of the electron density and mutually related as λ_1_ < λ_2_ < λ_3_. They can be used to calculate the ellipticity parameter, , which quantifies the amount of charge that accumulates preferentially. A large ε indicates topological instability and, as a result, an easily ruptured bond. The λ_3_ specifies how easily the BCP can be moved along the bond path,^208^ and the higher the value is, the stronger the interaction is. A local formulation of the virial theorem^208^ relates ∇^2^ρ(*B*C*P*) to electronic topological parameters by eq 23
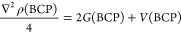
23where *G*(BCP) is the Lagrangian kinetic electron density, and *V*(BCP) represents the potential electron density (also known as the virial field).

Positive ∇^2^ρ(BCP) values indicate that *G*(BCP) is greater than *V*(BCP), implying that charge is being depleted along the bond path, as is typical of closed-shell interactions such as hydrogen bonding, ionic bonds, and van der Waals. Its negative values, on the other hand, indicate an excess potential energy at BCP in the form of internuclear charge concentration, which corresponds to covalent interactions; in this case, an electron density is localized in between two nuclei and is mutually accessible to both of them.^212^

Similarly, the −*G*(BCP)/*V*(BCP) ratio can be used for that purpose, because −*G*(BCP)/*V*(BCP) > 1 indicates that the intramolecular bond is closed and noncovalent, while 0.5 < −*G*(BCP)/*V*(BCP) < 1 points out that it is shared—for example partially covalent or ionic.^208,213,214^

Espinosa and coworkers^219,220^ demonstrated that interatomic interaction energy can be related to potential electron energy density at BCP using the following expression
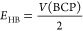
24The above relationship, according to Rozas et al.,^218^ allow hydrogen bonds to be classified as weak (*E*_HB_ < 12.0 kcal/mol), when ∇^2^ρ(BCP) > 0 and *H*(BCP) > 0, medium (12.0 < *E*_HB_ < 24.0 kcal/mol) if ∇^2^ρ(BCP) > 0 and *H*(BCP) < 0, or strong (*E*_HB_ < 24.0 kcal/mol) when ∇^2^ρ(BCP) < 0 and *H*(BCP) > 0, where *H*(BCP) denotes the density of electrons total energy, *G*(BCP) + *V*(BCP).

This is one of the most useful methods for calculating the energy of hydrogen bond interactions. Furthermore, Korth et al.^221^ demonstrated how to compute the relative intramolecular hydrogen bond enthalpy by comparing the sum of the conformer’s electronic and thermal enthalpies with intramolecular hydrogen bonds.

However, QTAIM analysis must to be used with caution in course of the studies. The molecule’s wavefunction, which is used to evaluated the aforementioned interactions, is determined by the functional and basis set used. As evidenced from refs (222) and (223), no relevant relationship between climbing Perdew’s Jacob’s ladder rungs and BCP densities was reported. The basis set, on the other hand, appears to be of primary concern. It has been demonstrated^224^ that small, double-ζ basis sets from Pople or Dunning’s families are insufficient to accurately assess the properties of BCP related to multiple and polar bonds, as well as weak hydrogen bond interactions. Instead, at least triple-ζ are recommended, which is plausible in the context of the current trend in their choice in the studies on antioxidants.

### Natural Bond Orbitals

One of the primary requirements for an antioxidant to effectively scavenge free radicals is that it becomes stable after the reaction. The spin density distribution throughout the molecule, which is often larger for conjugated systems, can be used to examine that property;^28,33,50,82^ however, natural bond orbital analysis^225−228^ represents a much more detailed investigation into the topic.

Refining the wavefunction into a Lewis-like structure corresponding to lone pairs and bonds gives an opportunity to track charge transfer by examining changes in the electron density at bonds, investigate hybridization of the orbitals and bonding interactions, as well as study delocalization and hyperconjugation effects.^10,34,61,67,85,103,123^ During the natural bond orbital analysis, the stabilization energy, *E*^(2)^, is derived for the electron transfer from filled donor orbital, *i*, to an empty acceptor orbital, *j*, and is related by eq 25
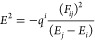
25where *F*_*ij*_ is the off-diagonal Fock matrix element and *q*^*i*^ the orbital occupancy, and *E*_*j*_ and *E*_*i*_ are diagonal elements. The intrepretion is clear —, the greater the *E*^2^ energy, the greater degree of interaction.

NBO analysis, just like QTAIM, also requires careful application. Alhough it is a straightforward and advantageous method, at the same it is heavily reliant on the geometry of the compound, which stems from the partitioning scheme of the electron density matrix and the localized nature of molecular orbitals. A simple, yet excellent, example of this can be found in the paper of Benassi and Fan^229^ where the authors reported on how the delocalization energy and orbital occupancy number differ in pyridine across its seven normal modes and small changes along the displacement coordinates. It has been demonstrated that even minor shift can result in significantly different *E*^(2)^ values, which is expected to be amplified in the case of larger antioxidant structures.

## Reactivity

### Thermochemistry

**Figure 3:**
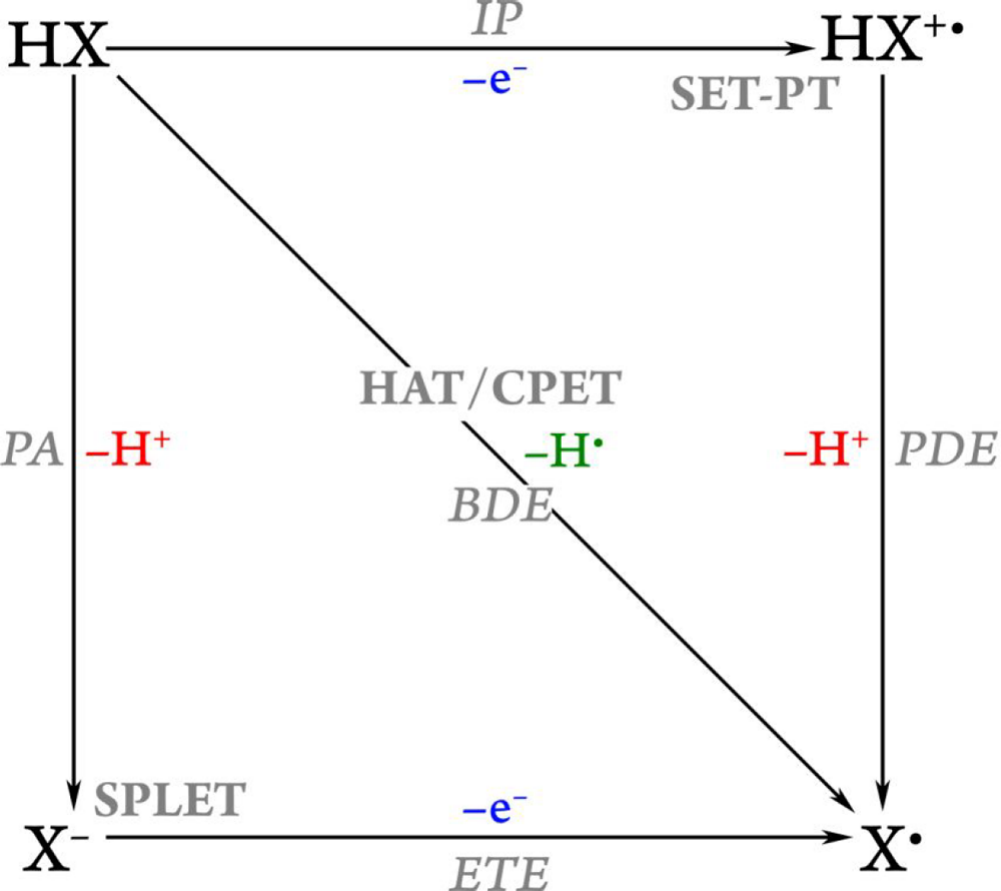
O’Farell–Jencks diagram of each single step involved in common reaction mechanisms.

In reality, the antioxidant’s ability to scavenge radicals is influenced not only by the antioxidant itself but also by the species with which it interacts. As a result, thermochemical calculations of known pathways (Table 4) produce far more useful data than intrinsic reactivity indices alone; one way to illustrate them is with O’Ferrall–Jencks diagrams^83,86^ (Figure 3). However, before proceeding, a foreword is required: hydroxyl radical, ^•^OH, is so reactive that it quickly reacts with almost any molecule in its vicinity at diffusion-limited rates, before an antioxidant can actually reach it. For this reason, it is skipped from computations and radicals with intermediate to low reactivity are chosen instead for assessing the antiradical potential.^230−232^ Peroxyl radicals, ROO^•^ (such as CH_3_OO^•^ or OOH^•^),^233,234^ are one of these because their half-lives are long enough that they can be intercepted before oxidizing biological targets.^235,236^ Although acid-base equilibrium of hydroperoxide equals 4.8, what means that O_2^•–^_ is typically present in physiological conditions, it is not very reactive species and the oxidation damage are primiarly steming from its protonated form.^267^ CCl_3_OO^•^ can also be considered because it is used in experiments to simulate larger radicals.^237^ Another possible reduction target might be the H_2_O_2_/O_2_ pair, which is more difficult to neutralize than other reactive oxygen species.^238^

**Table 4:** Naming, Associated Reactions, and Descriptions of the Most Commonly Studied Antioxidative Reaction Pathways

Name (typical acronym)	Related reaction	Description
Radical adduct formation (RAF)	Antox(OH)_*n*_ + X^•^ → [Antox(OH)_*n*_X]^•^	In a single step, the radical forms an adduct with the antioxidant, spreading the spin density across the newly formed molecule. The preferred reaction site is determined by the degree of the unpaired electron delocalization.
Hydrogen atom transfer (HAT)/Proton coupled electron transfer (PCET)	Antox(OH)_*n*_ + X^•^ → Antox(OH)_*n*−1_O^•^ + HX	A one-step mechanism in which an O–H bond is homiletically broken and a hydrogen atom is transferred from antioxidant to free radical, resulting in a more stable antioxidant radical. Low BDE values are common in compounds that promote this path. In the electrochemical sense, it is a reduction process. Although the products of HAT and PCET reactions are identical, the former involves the coordinated transfer of a proton and an electron as a single entity, whereas PCET involves the process of two separated particles, not necessarily from the same sets of orbitals. Formal HAT refers to chemical reactions that have not been defined as HAT or PCET.
Single electron transfer (SET)	Antox(OH)_*n*_ + X^•^ → Antox(OH)_*n*_^•+/•–^ + X^–/+^	Depending on the mutual IP and EA values, a single electron transfer occurs from an antioxidant to a radical or from a radical to an antioxidant. The deprotonation influences the thermochemical viability of the SET process to some extent.
Sequential electron transfer-proton transfer (SET-PT)		It occurs in two steps: first, a radical cation Antox(OH)_*n*_^•+^ is formed by electron transfer from an antioxidant to a free radical, and then, it deprotonates to form Antox(OH)_*n*−1_O^•^ species. The first step is described by the IP values, while the second step is described by the PDE values.
1. Electron transfer	1. Antox(OH)_*n*_ + X^•^ → Antox(OH)_*n*_^•+^ + X^–^	
2. Proton transfer	2. Antox(OH)_*n*_^•+^ → Antox(OH)_*n*−1_O^•^ + H^+^	
Sequential proton loss-electron transfer (SPLET)		The mechanism is divided into two steps: first, an antioxidant is deprotonated (as described by PA), and then, an electron transfer occurs from the deprotonated antioxidant to a free radical (described by IP). Because p*K*_a_ values influence the amount of deprotonated species in aqueous solution, knowing their number *a priori* can assist in determining the relative importance of this process.
1. Proton loss	1. Antox(OH)_*n*_ → Antox(OH)_*n*−1_O^–^ + H^+^	
2. Electron transfer	2. Antox(OH)_*n*−1_O^–^ + X^•^ → Antox(OH)_n-1_O^•^ + X^–^	
Sequential proton loss–hydrogen atom transfer (SPLHAT)		The mechanism is identical to SPLET, except that instead of an electron, a hydrogen atom is transferred in the second step. As a result, antioxidants containing at least two hydroxyl groups are particularly appealing. PA describes the first step, and BDE describes the second.
1. Proton loss	1. Antox(OH)_*n*_ → Antox(OH)_*n*−1_O^–^ + H^+^	
2. Hydrogen atom transfer	2. Antox(OH)_*n*−1_O^–^ + X^•^ → Antox(OH)_*n*−2_(O)_2_^•-^ + HX	

The polarity of the environment also has an effect on reaction energetics. To begin with, it should come as no surprise that reactions generating neutral species, such as RAF or HAT, perform better in nonpolar solvents than reactions that produces ions. This is due to the fact that nonpolar media do not provide enough solvation to stabilize charged species through the charge separation, thereby propelling the reaction forward. In consequence, such reactions are unlikely to occur in a significant number, and conventional studies in nonpolar media focus solely on RAF and HAT, with the remaining pathways being completely ignored.^60,63,106,121,139,143^

Furthermore, the first step in a multistep mechanism is thermodynamically significant and so determining its energetics allows for assessing reaction’s feasability, simplifying the analysis by rejecting unfavorable pathways. Because BDE, IP, and PA are the primary indices of the HAT, SET-PT, and SPLET,^239−242^ they can be used for this purpose.

Moreover, because SPLET mechanism is initiated by proton dissociation, which proclivity is controlled by the environment’s pH and acid–base equilibrium, the SET and SPLET processes are extremely closely coupled due to the spontanous. If molar fractions are being considered at the outset, the second step of SPLET actually controls an antioxidant’s reactivity, and in this case the entire mechanism becomes equivalent to SET; namely, it is identical to the SET reaction for an acid–base species with *N* – 1 protons. Herein, I will just mention that it is also an electrostatic potential map, which is a useful tool for distinguishing between electrophilic and nucleophilic centers, highlights positively charges hydroxyl hydrogen to be likely involved in proton dissociation mechanisms.^28,68^

Finally, because IP and EA values govern electron flow between antioxidant and radical, they can be used to estimate the direction of the SET mechanism, providing an early picture of the process. In general, the bare minimum for SET reactions with electrophilic radicals is IP(antioxidant) < EA(radical) and opposite holds true for the nucleophilic ones. In case of not so easily recognizable species, a general rule of knowing that electrons flow from the structure of lower IP to the structure of greater EA, makes it possible to predict which molecule will undergo oxidation and which will undergo reduction, and the reverse path can be thereby ignored.

One final point to mention about thermochemical calculations is that they require the Gibbs free energies of electron, proton, and hydrogen. The very last can be estimated directly at the applied level of theory, but the proton and electron cannot, at least not in a straightforward manner, necessitating the use of widely varying reference values.^178,180,181,243^

### Kinetics

An antioxidant is any substance that, even at low concentrations, significantly delays or prevents the oxidation of radical target. Therefore, it must not only react spontaneously with the oxidizing agent, but it must also react faster than the target it is designed to protect.^244,245^ This aspect can only be modeled using kinetic studies, which account for facets skipped by thermochemical studies—tunneling effects, weighted contribution of different mechanisms and different species to total antioxidative potential, or adherence to the Bell–Evans–Polanyi principle^3^—and is thus critical for accurately assessing antioxidative behavior.

Although spontaneity is an important criterion for chemical reactivity, it is not always enough because an exergonic reaction can occur at either fast or slow rates. When drawing conclusions from thermochemical data without considering kinetics, the Bell–Evans–Polanyi^246,247^ principle, which states that the most exergonic processes have the lowest activation energies and are thus kinetically favored, is implicitly assumed to be followed.^3^ On the other hand, ignoring a reaction path due to difficulties in locating a transition state may result in a more significant error than accepting the given rule wihout its confirmation, especially if it was discovered to hold true for a structurally similar compounds.^118^ It has been recently evidenced that Gibbs free energy is actually proportional to the activation energy of hydrogen atom transfer, and hence reaction rate of this mechanism.^90,268,269^

Endergonic channels do not need to be included in kinetic calculations in general because even if they occur at high rates, they are reversible to the point where no products are detected. However, moderately endergonic processes (typically with ΔG° < 10.0 kcal/mol) may still contribute to antioxidant capacity and should be addressed, particularly if their products evolve into other species quickly, providing a driving force, or the reaction barriers are low.^182^ This is the case for some RAF reactions,^82^ but it is particularly common for SET, where thermochemical and kinetic data may show opposing trends; in some cases, the mechanism may be associated with positive Gibbs energies and still be relevant, as has been demonstrated in a number of previously reported studies.^36,46,73,82,154^ Highly exergonic SET reactions involving donors with very low IP (such as monoanionic or polyanionic species) may, on the other hand, be found in the inverted region of the Marcus parabola, where that reaction barriers increase as Δ*G*° decreases what is often to be found for Gibbs free energies much lower than negative of reorganization energy.^248−250^ Despite this is an unexpected behavior, it underlines that particles with extremely low IP are unlikely to be efficient free radical scavengers.^46,101,125^ That is why using ionization potentials may be misleading, and electron-donating power or electrophilicity is far superior because, while it also relies on IP, it does so in a nonlinear fashion, with the shape of this dependency resembling the Marcus parabola.^46,101,112^

The activation barrier of a reaction is determined by the energy difference between the transition state and the reactants. However, in the case of electron-related processes, assessing it is not so straightforward. The barrier of electron transfer reaction (Δ*G*_ET_^≠^) is calculated in a different way, using the Marcus theory^250−252^ (eq 26), which defines it in terms of the reaction adiabatic free energy (Δ*G*_ET_^0^) and nuclear reorganization energy (λ)
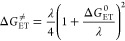
26

The reorganization energy is calculated as the difference between the vertical (Δ*E*_ET_) and adiabatic free energies of reaction and accounts for the orbitals relaxation
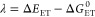
27

The reaction rate constants (*k*) can be calculated using the conventional transition state theory (TST) which is one of the most robust theoretical methodologies for this purpose, requiring only structural, energy, and vibrational frequency information for reactants and transition states, allowing it to be applied to a wide range of chemical processes.^253−255^ Despite its simplicity, it has been shown to reproduce fine the experimentally measured data on free radicals scavenging kinetics.^82,84,96,129^ It is computed usually in the framework of 1 M standard state^255^ using the Eyring equation (eq 28)
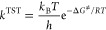
28where *k*_B_ and *h* are the Boltzmann and Planck constants, respectively. Δ*G*^^⧧^^ is the free energy of activation, calculated as the difference in energies between transition state and reactants, while *R* and *T* denote the gas constant and temperature, respectively.

The more sophisticated Eckart approach,^256^ also known as the zero-tunneling method, employs the Boltzmann average of the ratio of quantum and classical probabilities^257^ and is suggested for processes in which reactants are transformed into products over energy barriers. Such processes include the HAT reaction, which involves the motion of a light particle (here H^•^) that can easily tunnel, as well as some RAF pathways. Tunneling corrections (κ(*T*)), also known in the literature as transmission coefficients (γ(*T*)), are included, as is reaction path degeneracy (σ). κ(*T*) can significantly modulate reaction rate values between relatively similar reacting species, such as CH_3_OO^•^ or OOH^•^.^118^ It can be estimated using external software, for example Eyringpy.^258^ The number of identical reaction paths, on the other hand, is reflected by σ and can be calculated by labeling all similar atoms and counting the number of different but equivalent configurations that can be formed by rotating, but not reflecting them. They can also be established using the Pollak and Pechukas^259^ scheme (Table 5). Taking everything into account, eq 29 is obtained
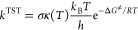
29

**Table 5:** Point Groups and the Reaction Path Degeneracy Values That Correspond to Them

Point group	σ	Point group	σ
C_1_	1	D_3h_	6
C_s_	1	D_5h_	10
C_2_	2	D_∞h_	2
C_2v_	2	D_3d_	6
C_3v_	3	T_d_	12
C_∞v_	1	O_h_	24
D_2h_	4		

As previously stated, radicals with high reactivity often react at diffusion-limited rates (*k* ≥ 10^8^ M^–1^ s^–1^) with the vast majority of chemical compounds. For an irreversible bimolecular diffusion-controlled reaction, the Collins–Kimball theory,^260^ in conjunction with the steady-state Smoluchowski rate constant^261^ and the Stokes–Einstein approaches,^262^ must be used to calculate rate constants properly (Table 6). They are frequently applied in case of RAF mechanisms, which is not surprising given that these types of radical attacks usually occur in the absence of energy barriers, making it difficult to localize the transition state.^82^

**Table 6:** Collins–Kimball Theory, Steady-State Smoluchowski, and Stokes–Einstein Equation Mathematical Formulations

Name	Related formula	Variables
Collins–Kimball theory	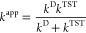	*k*_app_: apparent rate constant
		
Steady-state Smoluchowski	*k*^D^ = 4*πRD*_AB_*N*_A_	*k*^D^: steady-state Smoluchowski
		*k*^TST^: thermal rate constant (obtained from TST)
		*R*: reaction distance
		*N*_A_: Avogadro number
		*D*_AB_: mutual diffusion coefficient of the reactants A (free radical) and B (antioxidant)
		
Stokes–Einstein equation	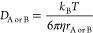	*k*_B_: Boltzmann constant
		*T*: temperature
		η: viscosity of solvent
		*r*: radius of solute

The total reaction rate constant values (*k*^TOT^) for all acid–base species (*i*) present at the specified pH multiplied by the corresponding molar fractions (*f*) allow the overall reaction rate constant (*k*^overall^), which corresponds to the empirically observed reaction rate, to be calculated
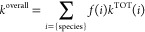
30

The reaction rate constants for each antioxidative mechanism (*j*) are then added to calculate the *k*^TOT^ values for all acid–base species
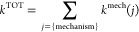
31

The *k*^mech^ (eq 32) is defined as the sum of reaction rate constants (*k*^TST^ or *k*^app^, depending on the kinetic model used, here simply represented by *k*) from to the same antioxidative mechanism but calculated at different reaction sites (*l*)
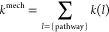
32

The branching ratio (Γ(*l*), (eq 33) can be used to calculate the percentage contribution of an antioxidative pathway (*l*) to the total reaction rate (*l*) using the following formula
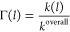
33

Furthermore, the relative antioxidative activity of the examined molecule can be calculated by dividing its *k*^overall^ by the *k*^overall^ of ref (45) (e.g., Trolox, Tx) as presented in eq 34
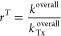
34

A threshold of *k*^overall^ = 1.2 × 10^3^ M^–1^ s^–1^ was proposed for quantifying antioxidant activity as a value close to the rate constant of the interaction between HOO^•^ and polyunsaturated fatty acids^3,21,126^. Compounds with higher *k*^overall^ values are thought to be effective antioxidants, while those with lower are thought to be ineffective.

### Thermochemistry and Kinetic Ensemble

Galano, Alvarez-Idaboy and coworkers combined the above considerations into the quantum mechanics-based test for overall free radical scavenging activity (QM-ORSA) protocol,^3,154,263^ which is proposed as a feasible tool to assess radical scavenging activity in physiologically relevant solvents. It entails the impact of all existing acid–base forms, identifying RAF, HAT, and SET mechanisms viabiality, and then subjecting them to kinetic analysis. This method has been already used successfully in numerous papers.^41,45,58,69,82,88,104,118^

## Concluding Remarks and Additional Considerations

Antioxidants were and are extensively studied using computational quantum chemistry. A number of useful tools are developed to provide insight into their electronic structure and reactivity. Importantly, scientists are not limited in their research to already known procedures but continously suggest new ones and apply them in their research. Therefore, each paper proposes some novel techniques in this matter, as well as new conclusions that provide greater insight into the chemistry of the antioxidants that have been already studied or projected from the scratch. As previously stated, because all antioxidants tend to exhibit similar patterns of activity, they can all be examined in the same way, whether we are talking about fullerenes, natural plant products, or entirely newly synthesized structures.

Certain points, however, should be reconsidered or reinterpreted:

- Almost all of the research considered reactive oxygen species, with only a minor focus on radicals containing nitrogen, carbon, and sulfur elements. These are known to be linked to oxidative stress,^264−266^ so research into them would fill the gap.
- When discussing *in vivo* activity, although solvation effects and hence deprotonation are considered, the importance of metabolites should be emphasized. The gut microbiota are the first to alter antioxidant structure, influencing primarily their absorption profile. They are then metabolized in the liver, where they undergo further changes that result in products with vastly different activity. By focusing solely on generic structures the outcomes are indeed limited to *in vitro* results, casting doubt on the physiological activities as free radicals scavengers, capable of halting the development of oxidative stress and resulting diseases.
- The dual nature of free radicals extends to the products derived from the antioxidants. Castañeda-Arriaga et al.^102^ discussed possible prooxidant behavior of such, in which the new forms may still be capable of oxidizing biologically relevant structures. The authors also proposed them to be able to self-regenerate, allowing them to work multiple times. This fresh topic appears to be a relevant aspect that needs to be considered when attempting to prove experimental observations.

## Data and Software Availability

Multiwfn (current version 3.7) is a free, open-source software developed by Tian Lu for wave function analysis and visualization. It is available for download at http://sobereva.com/multiwfn/. Eyring.py (current version 2.0) is a Python-based program developed by the Merino et al. group for computing rate constants of unimolecular or bimolecular reactions in the gas phase and in solution using transition state theory. For that purpose, it takes into account reaction symmetry, tunneling corrections, Collins–Kimball theory, Marcus theory, and species molar fractions. It is available at https://www.theochemmerida.org/eyringpy. GROMACS (current version 2021.5) is a free package, available under a LGPL license, created to perform molecular dynamics of proteins, lipids, nucleic acids as well as nonbiological systems. It is accessible from http://www.gromacs.org. Amber (current version 20) is a paid versatile suite of biomolecular simulation programs. It can be found at https://ambermd.org/index.php. CHARMM is a molecular simulation program that uses enhanced sampling methods and multiscale techniques to study many-particle systems. For academic users, the version “charmm” is free. The software is available for free download at https://academiccharmm.org.
